# Lactate Dehydrogenase Levels in the Saliva of Cigarette and E-Cigarette Smokers (Vapers): A Comparative Analysis 

**DOI:** 10.31557/APJCP.2021.22.10.3227

**Published:** 2021-10

**Authors:** Anitha Krishnan Pandarathodiyil, Anand Ramanathan, Ranjana Garg, Jennifer Geraldine Doss, Fazliny Binti Abd Rahman, Wan Maria Nabillah Ghani, Saman Warnakulasuriya

**Affiliations:** 1 *Department of Oral Diagnostic Sciences, Faculty of Dentistry, SEGI University, Jalan Teknologi, Kota Damansara, Petaling Jaya, Selangor, Malaysia. *; 2 *Department of Oral & Maxillofacial Clinical Sciences, Faculty of Dentistry, University of Malaya, Kuala Lumpur, Malaysia. *; 3 *Oral Cancer Research & Coordinating Centre (OCRCC), Faculty of Dentistry, University of Malaya, Kuala Lumpur, Malaysia. *; 4 *Department of Community Oral Health & Clinical Prevention, Faculty of Dentistry, University of Malaya, Kuala Lumpur, Malaysia. *; 5 *Faculty of Dentistry, Oral and Craniofacial Sciences, King’s College London, United Kingdom. *; 6 *WHO Collaborating Centre for Oral Cancer, United Kingdom.*

**Keywords:** LDH, saliva, vapers, smokers, enzyme, e-cigarettes

## Abstract

**Background:**

We examined the lactate dehydrogenase (LDH) enzyme levels in the saliva of vapers (e-cigarette users) and compared the data with cigarette smokers and a control group of non-smokers and non-vapers.

**Methods:**

Subjects were recruited among those responding to a social media announcement or patients attending the SEGi Oral Health Care Centre between May and December 2019, and among some staff at the centre. Five ml of unstimulated whole saliva was collected and salivary LDH enzyme activity levels were measured with a LDH colorimetric assay kit. Salivary LDH activity level was determined for each group and compared statistically.

**Results:**

Eighty-eight subjects were categorized into three groups (control n=30, smokers n=29, and vapers n=29). The mean ± standard deviation (SD) values for salivary LDH activity levels for vapers, smokers, and control groups were 35.15 ± 24.34 mU/ml, 30.82 ± 20.73 mU/ml, and 21.45 ± 15.30 mU/ml, respectively. The salivary LDH activity levels of smoker and vaper groups were significantly higher than in the control group (p = 0.031; 0.017). There was no significant difference of salivary LDH activity level in vapers when compared with smokers (p= 0.234).

**Conclusion:**

Our findings showed higher LDH levels in the saliva of vapers when compared with controls, confirming cytotoxic and harmful effects of e-cigarettes on the oral mucosa.

## Introduction

Cigarette smoke contains over 4,000 different chemicals, 400 of which are proven carcinogens and 70 are carcinogenic to humans or animals (IARC, 2004). In addition, various oxidants and numerous oxygen free radicals, volatile aldehydes are found, that have a detrimental effect on biomolecules (Yeh et al., 2008) and percolates into saliva that bathes the oral mucosa (Johnson, 2001). Smoking is a rich source of oxidants. It is considered the main contributor to the increased production of Reactive Oxygen Species (ROS) which may overwhelm the capacity of antioxidant defence systems (Akpotuzor et al., 2012). 

The use of electronic cigarettes (e-cigarettes), commonly known as vaping, is becoming popular as they are deemed less harmful (Glasser et al., 2017). E-cigarettes come in various forms and are also known under different names including electronic nicotine delivery systems (ENDS), vapor pens, e-hookah, and vape popes. It is claimed that e-cigarettes offer a less harmful nicotine-delivery alternative to combustible cigarettes. E-cigarettes are capable of delivering nicotine without tobacco combustion (Glasser et al., 2017; Grana et al., 2014). The e-cigarettes heat a liquid mixture (e-liquid) containing propylene glycol or glycerin, nicotine, and other additives thereby producing a vapor (Grana et al., 2014). 

E-cigarettes do not produce various toxins such as carbon monoxide that are commonly associated with cigarette smoke due to the absence of tobacco combustion (Goniewic et al., 2014). However, tobacco-derived chemicals such as volatile organic compounds and nitrosamines (Goniewicz et al., 2014), heavy metals, and silicate particles from the device’s heating elements (Goniewicz et al., 2014; Williams et al., 2013) may be present in e-cigarette aerosols at low but potentially biological levels. 

Lactate dehydrogenase (LDH) is a cytoplasmic enzyme that is present in the cells of the human body. This enzyme catalyses the conversion of glucose into pyruvic acid during aerobic glycolysis. When oxidative stress or oxidative damage occurs in the body, LDH may be released thus raising its level in serum and saliva. The extracellular leakage of this enzyme indicates cell damage or cell death (Narang et al., 2001). LDH activity has been extensively studied in the literature in serum and tissue samples of tobacco smokers (Iglesias, et al., 2020). 

Salivary LDH concentrations as an expression of cell death can be considered a specific indicator of the effect of mucosal damage leading to the loss of integrity of the oral mucosa (Rao et al., 2017). Saliva is increasingly utilized as a biological fluid that is also important in the evaluation of overall health. 

Published literature has produced consistent results on the effects of smoking on the LDH activity but the levels vary depending upon the criteria used for sampling and the methods of analysis. In this study, we hypothesize that the smoke produced by vaping e-cigarettes has detrimental effects on oral tissues. To assess any damage, we selected to examine Lactate Dehydrogenase Enzyme levels in the saliva of vapers and to compare the data with that of cigarette smokers and in a group of non-smokers/non vapers. 

## Materials and Methods

*Participants*

Volunteers for the study were collected through different routes. Volunteers for the vaping group were mostly recruited through advertisements placed in Facebook and Whatsapp group messages and anyone volunteering was offered a free dental check-up. Cigarette smokers and controls were recruited among patients attending the SEGi Oral Health Care Centre for dental check-ups or treatments between May 2019 to December 2019. About 40 patients were seen daily at this centre. During initial screening, all patients were invited to participate in the study and given a patient information sheet which had the basic details about the study design (available from the authors on request). The criteria for smokers and vapers were that they should have practised the habit regularly for a minimum period of 6 months. The control group consisted of subjects who did not practise smoking or vaping. Anyone volunteering was interviewed by the study administrator who collected and recorded information on their tobacco and alcohol consumption and medical histories to exclude any subjects who were tobacco chewers, regular alcohol users, those with chronic illnesses or taking regular medications. Any volunteers for the control group were also selected among non-smokers and non-vapers from the staff at the centre and similar exclusion criteria were applied. All received full mouth examinations to exclude any one with an oral potentially malignant disorder. Basic Periodontal Examination (BPE) was conducted to exclude subjects with BPE grades 3 or 4 in any sextant. Those qualifying for the study provided their informed consent and were given appointments and instructions to return to participate in the study. The volunteers were requested not to consume any food 2 hours prior to their appointment on the day of saliva collection. Based on the social histories ninety subjects were selected and categorized into three groups (controls, n=30, smokers, n=30, and vapers, n=30).

*Saliva Sample Collection*

An unstimulated saliva sample was collected from each volunteer; the samples were coded and categorized into three groups (smokers, vapers and controls,). During saliva collection, they were seated comfortably on the dental chair and asked to thoroughly rinse their mouths twice using distilled water. Following which, subjects were asked to allow pooling of saliva to accumulate in his or her mouth for 5 minutes (unstimulated whole saliva). The subjects were then asked to spit the accumulated whole saliva in to sterile, disposable, wide mouthed containers, until a minimum desired quantity of 5 ml was obtained. The saliva tubes were immediately transported on ice to the laboratory at the University Malaya for further analysis, where the samples were then centrifuged and stored at -20°C for further analysis.

*Biochemical Analysis*

The salivary LDH enzyme activity levels of the saliva samples were measured with a commercially available LDH Colorimetric Assay Kit (ab102526, Abcam, MA, US) according to the manufacturer’s protocol. In this assay, 50 μl of each subject’s saliva was added to 50 μl of LDH reaction mix in duplicate. The absorbance was measured at a wavelength of 450 nm at 37^o^C. The measurements were done in duplicate. The pH of the saliva was checked by pipetting out 1 ml of the collected saliva on to the pH strips. The mean level of salivary LDH activity was determined for each group and compared.

*Statistical Analysis*

Descriptive data are presented as means ± standard deviations, and percentages. Differences in sociodemographic characteristics and salivary pH levels among the three study groups were assessed by Mann-Whitney U and Kruskal-Wallis test for continuous data, while categorical data was compared using Pearson Chi square test. Receiver operating characteristics (ROC) analysis was conducted to determine the optimal cut-off points of LDH levels for the study groups. Univariate and multivariate logistic regression analyses were conducted to compare the LDH salivary levels among the three study groups. Statistical analysis was conducted with SPSS version 21.0 (SPSS Inc, Chicago, IL, USA). A p value of less than 0.05 (p < 0.05) was indicated statistically significant.

*Ethical Approval for the study*

Ethical approval for the study was obtained from SEGi University Ethics Committee [Medical Ethics Approval Code: SEGiEC/StR/FOD/32/2019-20] and Medical Ethics Committee, Faculty of Dentistry, University of Malaya [Medical Ethics Approval Code: DFOS2027/0096(L)].

## Results

There was a total of 90 subjects recruited for this study in the three groups, with 30 subjects in each group (smokers, vapers and controls). However, one subject each in the smoker and one in the vaper group were excluded due to a mismatch of their inclusion criteria found on reverification. A subject in the smoker group was excluded since he had been diagnosed with diabetes one month ago, and the subject in vaper group was excluded owing to a current history of smoking not revealed at the interview. Therefore, the final number of subjects in the control group was 30, whereas in the smoker and vaper group was 29 each and the total subjects analysed in this study were 88. 

 The socio-demographic characteristics of the study subjects are shown in Table 1. The age range of the subjects was from 19 to 70 years. The mean ± standard deviation (SD) of age was 31.57 ± 13.47 among healthy subjects, 32.31 ± 12.76 among smokers, and 26.00 ± 7.35 among vapers (p = 0.075, Kruskal-Wallis test). Majority of the subjects in smoker and vaper groups were males, whereas there were more females in the control group (p = 0.007, Pearson Chi-Square test). The smoker group consisted of cigarette smokers with the duration of smoking habit at minimum of 2 years, whereas the vaper group consisted of subjects with a vaping history at a minimum of 6 months’ duration. The mean salivary pH values in the study groups ranged from 7.17 to 7.24. It was found to be highest in the vaper group with a mean value of 7.28 (p = 0.790, Kruskal-Wallis test) suggesting a tendency for the saliva of vapors to be slightly more alkaline. 

**Figure 1:**
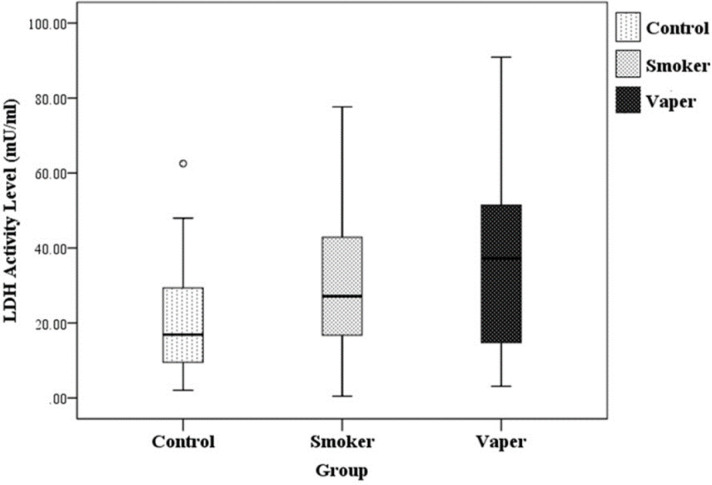
Salivary LDH Activity Levels in the Control, Smoker, and Vaper Groups

The salivary LDH activity levels for vaper, smoker, and control groups are illustrated in Figure 1. The mean ± standard deviation (SD) values for salivary LDH activity level for vaper, smoker, and control groups were 35.15 ± 24.34 mU/ml, 30.82 ± 20.73 mU/ml, and 21.45 ± 15.30 mU/ml respectively.

Univariate and multivariate analysis of the salivary LDH activity levels among the study groups are shown in Table 2. The salivary LDH activity level of smoker and vaper groups were significantly higher than in control group (p= 0.031; 0.017). However, there was no significant difference of salivary LDH activity level in vaper when compared with smoker group (p= 0.234).

The comparison of salivary LDH activity level with smoking and vaping habit duration was performed using Mann Whitney U test in the smoker and vaper groups, respectively. No significant difference was found between salivary LDH activity level and habit duration [p =0.164 (smokers); 0.194 (vapers)] (Table 3).

**Figure 2:**
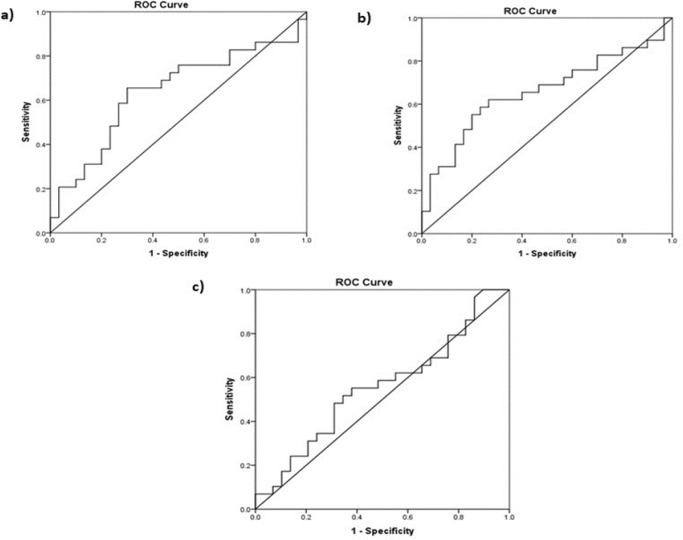
Shows (a) the area under the ROC curves (AUC) for smoker was 0.638 (95% CI: 0.492–0.784, p = 0.069), (b) the area under the ROC curves (AUC) for vaper was 0.659 (95% CI: 0.515–0.802, p = 0.036) and (c) the AUC to distinguish smoker and vaper group was 0.554 (95% CI: 0.404–0.703, p = 0.484)

Receiver operating curve (ROC) analysis was used to determine the optimal cut-off values of salivary LDH activity levels in the groups. The area under the ROC curves (AUC) for smoker and vaper were 0.638 (95% CI: 0.492–0.784, p = 0.069) (Figure 2a) and 0.659 (95% CI: 0.515–0.802, p = 0.036) (Figure 2b). The optimal cut-off levels were 23.64 mU/ml for smoker (sensitivity of 65.5% and specificity of 70.0%) and 25.65 mU/ml for vaper (sensitivity of 62.1% and specificity of 73.3%). Whereas, the optimal cut-off value to distinguish smoker and vaper group was 35.09 mU/ml (sensitivity of 55.2% and specificity of 62.1%) with AUC of 0.554 (95% CI: 0.404–0.703, p = 0.484) (Figure 2c).

**Table 1:** Socio-Demographic Characteristics of the Study Subjects

	Control (n=30)	Smoker (n=29)	Vaper (n=29)	p-value
Age (years)				
Mean ± SD	31.57 ± 13.47	32.31 ± 12.76	26.00 ± 7.35	**0.075** ^a^
Range	19-68	20-70	19-50	
Gender				
Males (%)	14 (46.67)	24 (82.76)	22 (75.86)	
Females (%)	16 (53.33)	5 (17.24)	7 (24.14)	**0.007** ^b^
Habit Duration (years)				
Mean ± SD	NA	10.83 ± 10.20	2.22 ± 1.87	**< 0.001** ^c^
Range	NA	2-50	0.5-7	

NA, not applicable; SD, standard deviation; bold, p-value < 0.05; ^a^, Kruskal-Wallis test; ^b^, Pearson Chi-Square test; ^c^, Mann-Whitney U test

**Table 2:** Univariate and Multivariate Analysis of Salivary LDH Activity Levels in the Smoker, Vaper, and Control Groups, Adjusted for Age and Gender

	LDH Activity Level		Univariate		Multivariate+	
Category	Low n(%)	High n(%)	OR (95%CI)	p-value	OR (95%CI)	p-value
Control	21 (70.0)	9 (30.0)	4.443 (1.485-13.234)	0.008	3.659 (1.127-11.882)	0.031
Smoker	10 (34.5)	19 (65.5)				
Control	22 (73.3)	8 (26.7)	4.5 (1.493-13.564)	0.008	4.271 (1.296-14.080)	0.017
Vaper	11 (37.9)	18 (62.1)				

**Table 3:** Comparison between Salivary LDH Activity Level and the duration of the Habit of Smoker and Vaper Groups

		Smoker (n=29)				Vaper (n=29)		
Variable		n	Mean ± SD (mU/ml)	p-value		n	Mean ± SD (mU/ml)	p-value
Habit duration (years)	< 5	10	22.79±14.61	0.164	<1	8	27.06±22.76	0.194
	≥ 5	19	35.04±22.52		≥1	21	40.92±25.38	

**Table 4:** Summary of Studies which Investigated LDH Levels in Serum only, Saliva only, & Serum and Saliva

Author [Ref no]	Study Groups	Body fluids examined	Results Mean/ Median of LDH levels (SD)	Summary findings
"Iglesias-Velázquez et al. [10]Oral Diseases, 2020; doi: 10.1111/odi.13630. Online ahead of print"	"No of studies: 13 for systematic review, 10 for meta-analysisOC (n=303) OL (n=80) OSF (n=60) Other OPMD (n=9) Controls (n=303)"	"Saliva"	"Standardized mean difference (SMD); 95%CIHigher LDH levels in OC than control: SMD 9.5; 95% CI 7.0–12.0 (S)Higher LDH levels in OL than control:SMD 11. 7; 95% CI 1.0–22.3 (S)Higher LDH levels in OSF than control:SMD 25.8; 95% CI 1.7–53.4 (NS)Higher LDH levels in OC than OL:SMD 5.62; 95% CI 2.1–9.1 (S)"	Salivary LDH levels were significantly higher in OC patients compared to OL, OSF and controls.
"Gholizadeh et al. [33]BMC Oral Health, 2020; 20:314"	"M: 34 F: 66OSCC (n=25), Mean age: 61.0±3.2OLP (n=25), Mean age: 49.7±3.2OLR (n=25), Mean age: 52.7±2.8Control (n=25), Mean age: 42.7±2.4"	"SerumUnstimulated SalivaStimulated saliva"	"Mean±SEOSCC: 335.3±41.1OLP: 52.4±14.8OLR: 122.3±16.6Control: 29.4±6.5OSCC: 99.8±49.3OLP: 4.9±1.3OLR: 14.7±3.0Control: 3.8±1.1OSCC: 112.2±40.2OLP: 3.6±1.0OLR: 20.9±5.5Control: 3.5±1.1"	Serum and salivary levels of LDH in OSCC patients were significantly higher than other groups. OLRs had higher serum levels of LDH than OLP and control.
"Panda et al. [19]Journal of Oral and Maxillofacial Pathology, 2020; 24(1):183"	"M: 108 F: 12Age range: 20-70OSF (n=40)OL (n=40)Control (n=40)"	"SerumUnstimulated saliva"	"Mean±SDOSF: 534.6±12.6OL: 288.7±13.5Control: 217.1±38.1OSF: 631.7±7.7OL: 492.8±16.7Control: 140.6±8.9"	Salivary and serum LDH levels in patients with OSF and OL is significantly higher compared to controls.
"Mantri et al. [34]The Journal of Contemporary Dental Practice, 2019; 20(8):970–973"	"Age range: 18-70OSCC (n=30)OSF (n=30)Tobacco chewer (n=30)Control (n=30)"	"Unstimulated saliva"	"Mean±SDOSCC: 592.1±28.6OSF: 350.4±5.9Tobacco chewer: 125.2±13.4Control: 86.1±7.1"	LDH levels were significantly higher in OSCC, OSF and healthy patients who were habitual tobacco chewers, as compared to normal controls.
"Kumar et al. [16]International Journal of Scientific Research, 2019; 8(4):4-6"	"M: 45 F: 30Active smoker (n=25), Mean age: 41.5Passive smoker (n=25), Mean age: 37.0Control (n=25), Mean age: 36.4"	"Unstimulated saliva"	"Mean±SDActive smoker: 433.0±330.8Passive smoker: 356.5±397.0Healthy control: 230.6±139.1"	Positive association between cigarette smoking (both active and passive) and elevated salivary LDH levels.
"Madhumitaa et al. [35]International Journal of Research in Pharmaceutical Sciences, 2018; 9(3): 853-856"	"OSCC (n=30)Control (n=30)"	"Serum"	"Mean±SD OSCC: 285.7±68.0Control: 121.2±17.7"	LDH levels was significantly higher in OSCC patients as compared to controls.
"Mishra et al. [36]Journal of International Society of Preventive and Community Dentistry, 2018; 8(4):289-295"	"M: 40 F: 0OSF (n=20), Mean age: 28.6±10.4Control (n=20), Mean age: 26.2±7.0"	"SerumUnstimulated saliva"	"Mean±SD OSF: 408.4±158.4Control: 313.1±82.7OSF: 1057.3±640.1Control: 668.3±498.5"	Serum and salivary LDH levels were significantly higher in OSF patients. Serum LDH is positively correlated with frequency of habit and mouth opening in OSMF patients.
"Rao et al. [37]International Journal of Dentistry Research, 2017; 2(2): 31-35"	"M: 36 F: 24OSCC (n=30), Mean age: 57.3±9.5Control (n=30), Mean age: 34.9±14.9"	"SerumUnstimulated saliva"	"Mean±SD OSCC: 540.5±88.8Control: 390.9±71.1OSCC: 906.4±239.5Control: 201.4±89.1"	LDH levels in both serum and saliva are significantly higher in OSCC patients.
"Nandita et al. [38]International Journal of Preventive Clinical Dentistry Research 2017;4(3):196-200"	"OSCC (n=10)OL (n=10)OSF (n=10)Control (n=10)"	"SerumUnstimulated saliva"	"Mean±SD OSCC: 886.3±138.9OL: 471.6±72.3OSF: 512.7±46.7Control: 251.5±48.3OSCC: 1126.0±194.5OL: 563.6±80.6OSF: 668.0±75.1Control: 376.1±76.5 "	Salivary and serum LDH levels were highest in oral cancer, followed by OSF and OL. LDH levels in these three groups are significantly higher than in controls.
"Mohan et al. [22]Journal of Medical Science and Clinical Research. 2017;5(2):17638-43."	"Control (n= 10)Tobacco users (n= 10) Potentially malignant disorders PMD (n= 100"	"SerumUnstimulated saliva"	"Mean±SD Control: 422.20 ±92.53Tobacco users: 495.60± 123.49PMD: 3,516.80 ±1,297.30Control:426.70± 216.51Tobacco users: 677.90 ±235.87 PMD: 2,470.60 ±938.20"	There is significant difference in salivary LDH level between healthy controls and tobacco users subjects and also healthy controls and potentially malignant disorders subjects.
"Swamy & Ganiger [39]International Journal of Otorhinolaryngology & Head and Neck Surgery, 2016; 2(4): 234-237"	"OSF (n=40), Mean age: 49.5±11.7Control (n=40), Mean age: 46.2±10.3"	"Serum"	"Mean±SD OSF: 492.2±16.4Control: 117.2±19.5"	LDH levels in OSF patients is significantly higher than in controls.
Author [Ref no]	Study Groups	Body fluids examined	Results Mean/ Median of LDH levels (SD)	Summary findings
"Lokesh et al. [23]Journal of Clinical and Diagnostic Research, 2016; 10(2):ZC34-37"	"Age range: 35-65OSCC (n=30)Control (n=20)"	"Unstimulated saliva"	"Mean±SD OSCC: 1225.4±221.8Control: 497.0±51.8"	LDH levels were significantly higher in patients with OSCC. LDH values increased proportionally in relation to the histopathological grade of tumour.
"Kallalli et al. [40]Journal of Oral Pathology & Medicine, 2016; 45(9):687-690"	"Age range: 20-70OSCC (n=25)OSF (n=25)Control (n=10)"	"Unstimulated saliva"	"Mean±SD OSCC: 631.0±39.8OSF: 608.3±30.2Control: 182.2±34.9"	LDH levels were significantly higher in patients with OSCC and OSF.
"Rathore et al. [41]Journal of Indian Academy of Oral Medicine and Radiology, 2015: 27(1): 29-34"	"M: 109 F: 11OSCC (n=30)OL (n=30)OSF (n=30)Control (n=30) "	"Serum"	"Mean±SD OSCC: 323.8±46.8OL: 277.9±33.3OSF: 249.7±44.7Control: 161.9±36.1"	LDH levels were highest in OSCC patients, followed by OL and OSF. As compared to controls, LDH levels was significantly higher in patients with OSCC, OL and OSMF.
"Patel & Metgud [42]Journal of Cancer Research and Therapeutics, 2015; 11(1):119-23"	"OSCC (n=25)OL (n=25)Control (n=25)"	"Unstimulated saliva"	"Mean±SD OSCC: 686.4±81.8OL: 497.0±100.4Control: 261.2±75.9"	LDH levels were significantly higher in OL and OSCC patients. LDH level increases with increasing histopathological grade of the tumour.
"D’Cruz & Pathiyil [43]South Asian Journal of Cancer, 2015; 4(2): 58–60"	"OSCC (n=30)Control (n=30)"	"Unstimulated saliva"	"Mean±SD Well-diff OSCC: 355.8±16.7Mod-diff OSCC: 484.2±25.8Poor-diff OSCC: 620.4±18.7Control: 117.3±19.4"	LDH levels were higher in OSCC as compared to controls. LDH level increases with increasing histopathological grade of the tumour.
"Joshi & Golgire [44]Journal of Oral & Maxillofacial Pathology, 2014; 18(Suppl 1): S39–S44"	"OSCC (n=30), Mean age: 48.0OL (n=30), Mean age: 41.1Control (n=30), Mean age: Age matched"	"Unstimulated saliva"	"MeanOSCC: 788.7OL: 519.4Control: 267.2"	LDH levels was significantly increased in OSCC and OL as compared to controls.
"Rai et al. [17]Adv Med Dent Sci. 2007; 1(1):1-4."	"Age range: 28-57 Smokers with periodontitis (n=32)Non-smokers with periodontitis (n=31)Smokers with healthy periodontium (n=28)Non-smoker with healthy periodontium (n=22)"	"Saliva"	"Mean±SDNon-smokers without periodontitis, 382.2±16.2 Smokers without periodonttis 412.3±16.3 Non-smokers with periodontitis 422.1±17.2Smokers with periodonttis 472.1±18.9"	Salivary levels of LDH were significantly higher in smokers with periodontitis as compared to others.

## Discussion

LDH is an enzyme present in the cytoplasm of almost every cell in the human body. It catalyses the conversion of lactate to pyruvate during anaerobic glycolysis. It is well-established that the main source of LDH enzyme in saliva is from the oral epithelium (Nagler et al., 2001). Cellular necrosis, cell death, and tissue breakdown are events that causes LDH to be secreted extracellularly, thus causing an increase in the level of extracellular LDH (De La Pena et al., 2007; Sivaramakrishnan et al., 2015). Hence, it is only logical to associate an increased level of the enzyme in saliva in the presence of oral epithelial cellular necrosis, breakdown, and damage (Nagler et al., 2001) and it has been proposed that salivary LDH may be used to detect subtle oral mucosal pathologies (Nagler et al., 2001).

Utilizing saliva as a liquid biopsy medium to study levels of biomarkers in the body is gaining popularity. A recent review of literature confirmed that salivary proteins have the potential to be used as biomarkers for head and neck cancer (Amenábar et al., 2020). The collection of samples could be easy, cost effective, and non-invasive. However, it is surprising to note that only a few studies have investigated the LDH enzyme levels in saliva in smokers (Table 4) (Rao et al., 2017; Kumar et al., 2019; Rai et al., 2007). Since ours is the first study that has investigated the salivary LDH levels in vapers, no baseline information is available on this from prior studies. There have been studies, however, on the salivary LDH levels in healthy subjects (De La Pena et al., 2007) and among smokers (Rao et al., 2017). In addition, LDH activity has been previously investigated as an early detection tool or biomarker for various diseases in the oral cavity such as periodontal disease (De La Pena et al., 2007; Rai et al., 2007), oral leukoplakia (Shetty et al., 2012; Panda et al., 2020), oral submucous fibrosis (Panda et al., 2020; Mishra et al., 2018) and oral squamous cell carcinoma (Shetty et al., 2012). An earlier study (ArRejaie et al., 2019) reported vaping as posing a risk to periodontal tissue health and induced oxidative stress leading to the release of destructive inflammatory cytokines which further caused damage to the periodontium. LDH levels were also reported to be elevated in subjects with potentially malignant disorders (Mishra et al., 2018; Mohan et al., 2017) as well as squamous cell carcinomas of the oral cavity (Lokesh et al., 2016). A systematic review on the oral health impact of electronic cigarette suggested a wide range of oral health sequelae as a result of this habit (Yang, et al., 2020). 

Salivary LDH levels studied in smokers have elucidated some interesting findings (Rao et al., 2017; Rai et al., 2007), several reporting statistically significant differences observed between active smokers and healthy controls. Studies by Rao (2017) and Rai (2007), reported higher levels of salivary LDH in smokers when compared to non-smokers. Mohan (2017) found increased serum and salivary LDH activity in both tobacco users and subjects with potentially malignant disorders in comparison with normal controls. Our results concur with the results from these previous studies, showing significantly higher levels in the salivary LDH among smokers than the non-smoking control group. 

Smoking produces reactive oxidative substances (ROS) (Huang et al., 2005). ROS has been reported to induce damage to keratinocytes lining the mouth and the airways, activate oxidative-sensitive cellular pathways, and induce DNA damage (Valavanidis et. al., 2009). The increased salivary LDH levels among smokers as compared to non-smokers could arise from the breakdown of oral epithelial cells due to the presence of ROS.

To our knowledge this is the first instance, a study to measure the salivary LDH levels of vapers has been attempted. In our study, the salivary LDH levels in e-cigarette smokers or vapers showed an increased level when compared to the control and the smokers’ group, whereby the vaping population recorded the highest salivary LDH levels. 

The evidence presented here, that vapers had the highest salivary LDH levels indicates higher cell death and oral epithelial cell breakdown, thus shedding some light on its potential health risks. Furthermore, based on the cut-off points determined by ROC analysis in our study, vapers and smokers were four times and almost four times (respectively) more likely to have high LDH levels than non-smokers or non-vapers, reiterating potential harmful effects from both these habits. 

Although ENDS has been advocated to be significantly less harmful than combustible tobacco (Drope et al., 2017) and provide an alternative method for smoking cessation (Rom et. al., 2015), to some extent, ENDs vapour content is regarded to be more harmful than conventional cigarettes as it contains various aldehydes including acrolein (IARC, 2021) and formaldehyde which results in damage to DNA and delays and impaired wound healing (Sundar et al., 2016). Thus, increased uptake of vaping by the younger generation would expose them to undue health risks, systemic and oral in nature, at an earlier age. Furthermore, concerns have been raised that e-cigarettes might act as a gateway for future smoking especially among teenagers, as well as a means to normalise smoking (Alawsi et al., 2015; Barrington-Trimis et al., 2016). In light of emerging evidence regarding potential harmful effects of vaping, it is imperative for early education to be disseminated to adolescents and young adults to correct any misconception about vaping in comparison to smoking habit and the potential health risks related to both these addictive habits. This is evident by the comparable salivary LDH levels between vapers and smokers in this study.

The strength of our research would be that it is a pioneer study that has attempted to record the salivary LDH activity levels in a vaping population and compare them with that of smokers and normal controls. The non-invasive, and cost-effective technique employed by means of utilizing saliva to investigate the enzyme level is another strength. The weakness of our study would be that the serum LDH levels were not investigated and compared with the salivary levels. This was due to the concern that the invasive nature of such a procedure may dissuade volunteers from participating in the study. Our research findings will guide future studies in evaluating the impact of e-cigarettes on the oral mucosa especially in youth, which will ultimately help to inform the public and the policy makers about the potential health risks of e-cigarettes. 

In conclusion, while evidence to date reveals that e-cigarettes release fewer toxins and carcinogens than conventional cigarettes, this study found significantly raised salivary LDH levels among vapers. As knowledge gaps still exist in terms of long-term exposure effects of vaping and its integration with toxicity assessments, more data is urgently needed to understand the potential health risks and public health impact of vaping.

## Author Contribution Statement

Conceptualization: Anitha Krishnan P, Anand Ramanathan, Ranjana Garg; Methodology: Anitha Krishnan P, Wan Maria Nabillah Ghani, Anand Ramanathan; Results analysis: Jennifer Geraldine Doss, Wan Maria Nabillah Ghani, Fazliny Abd Rahman, Saman Warnakulasuriya ; Writing: Anitha Krishnan P, Anand Ramanathan, Ranjana Garg, Wan Maria Nabillah Ghani, Jennifer Geraldine Doss, Saman Warnakulasuriya; Review: Anitha Krishnan P, Anand Ramanathan, Ranjana Garg, Wan Maria Nabillah Ghani, Jennifer Geraldine Doss, Saman Warnakulasuriya
